# The epidemiological characteristics of enterovirus infection before and after the use of enterovirus 71 inactivated vaccine in Kunming, China

**DOI:** 10.1080/22221751.2021.1899772

**Published:** 2021-03-30

**Authors:** Hongchao Jiang, Zhen Zhang, Qing Rao, Xiaodan Wang, Meifen Wang, Tingyi Du, Jiaolian Tang, Shuying Long, Juan Zhang, Jia Luo, Yue Pan, Junying Chen, Jing Ma, Xiaomei Liu, Mao Fan, Tiesong Zhang, Qiangming Sun

**Affiliations:** a Institute of Medical Biology, Chinese Academy of Medical Sciences and Peking Union Medical College, Kunming, People’s Republic of China; b Kunming Children’s Hospital, The Affiliated Children’s Hospital of Kunming Medical University; Institute of Pediatric Disease Research in Yunnan, Kunming, People’s Republic of China; c Yunnan Key Laboratory of Vaccine Research & Development on Severe Infectious Diseases, Kunming, People’s Republic of China; d Yunnan Key Laboratory of Children’s Major Disease Research, Kunming, People’s Republic of China; e State Key Laboratory of Respiratory Disease, National Clinical Research Center for Respiratory Disease, Guangzhou Institute of Respiratory Health, the First Affiliated Hospital of Guangzhou Medical University, Guangzhou, People’s Republic of China

**Keywords:** Hand-foot-and-mouth disease, enterovirus A71 vaccine, surveillance, epidemiological characteristics, pathogen spectrum

## Abstract

Enterovirus A71 (EV-A71) inactivated vaccines have been widely inoculated among children in Kunming City after it was approved. However, there was a large-scale outbreak of Enteroviruses (EVs) infection in Kunming, 2018. The epidemiological characteristics of HFMD and EVs were analysed during 2008–2018, which are before and three years after EV-A71 vaccine starting to use. The changes in infection spectrum were also investigated, especially for severe HFMD in 2018. The incidence of EV-A71 decreased dramatically after the EV-A71 vaccine starting use. The proportion of non-CV-A16/EV-A71 EVs positive patients raised to 77.17–85.82%, while, EV-A71 and CV-A16 only accounted for 3.41–7.24% and 6.94–19.42% in 2017 and 2018, respectively. CV-A6 was the most important causative agent in all clinical symptoms (severe HFMD, HFMD, Herpangina and fever), accounting from 42.13% to 62.33%. EV-A71 only account for 0.36–2.05%. In severe HFMD, CV-A6 (62.33%), CV-A10 (11.64%), and CV-A16 (10.96%) were the major causative agent in 2018. EV-A71 inactivated vaccine has a good protective effect against EV-A71 and induced EVs infection spectrum changefully. EV-A71 vaccine has no or insignificant cross-protection effect on CV-A6, CV-A10, and CV-A16. Herein, developing 4-valent combined vaccines is urgently needed.

## Introduction

Enterovirus (EVs) belong to the family of picornaviridae, including poliovirus, echoviruses, coxsackieviruses, numbered enteroviruses, and rhinoviruses. Non-specific febrile illness, encephalitis, paralysis, HFMD, neonatal sepsis-like disease, and respiratory diseases generally induced by these diverse viruses [1]. HFMD is a communal infectious disease in children. The incidence of HFMD is mainly in children under five years of age [2]. The incidence of HFMD in infants under 6 months is lower than that in infants over 6 months, because of the protection of foetal antibodies, and it has increased gradually since 6 months of age. Children aged 1–2 are at the highest risk. EVs are major contributors to HFMD worldwide, with a wide range of clinical features varying from very mild to fatal infections [3]. HFMD can be induced by at least 20 serotypes of EVs in group A, among which EV-A71, coxsackievirus A16 (CV-A16), coxsackievirus A6 (CV-A6), and coxsackievirus A10 (CV-A10) are the most common, and most severe and fatal diseases are caused by EV-A71 infection.

HFMD is highly contagious and transmitted by fecal–oral, which primarily caused by human enterovirus A (HEV-A) and induced millions of infants and children to suffer from this disease in the Western Pacific every year [4]. Every year in China, April to June is the peak season of the HFMD epidemic. In some places, the autumn peak of the HFMD epidemic occurs from October to November, especially in the south China. In the past two decades, HFMD has been primarily prevalent in Asian countries, including China (mainland, Taiwan, Hong Kong), Malaysia, Japan, Singapore, Vietnam, South Korea, Thailand, and Cambodia. China is the country with the most reported morbidity and mortality of HFMD in the world. Besides, HFMD is a growing public health concern that poses a considerable disease burden and economic impact in affected areas [5,6]. Among the 9 million HFMD cases reported in China from 2008 to 2013, EV-A71 and CV-A16 were the primary pathogens [7]. In addition, numerous HFMD outbreaks in Asia, America, and Europe also were principally induced by these emerging viruses since 2010 [8–11]. Enterovirus generally induced HFMD in children and was previously prevailingly caused by EV-A71 and CV-A16. Of all HFMD cases reported to the World Health Organization (WHO) in 2010–2014, China accounted for 87.00% (9.8 million/11.3 million) [12]. However, recently, CV-A6 and CV-A10 have partially replaced EV-A71 and CV-A16, becoming the principal pathogens causing HFMD.

In order to control the epidemic of HFMD, the world’s first EV-A71 inactivated vaccine (human diploid cells), developed by the Institute of Medical Biology of the Chinese Academy of Medical Sciences, and was approved at the end of 2015. After that, EV-A71 vaccines have been widely inoculated among children in Kunming City, a medium-sized city with a resident population of 6.783 million in 2018, of which 1.08 million are children. Till the end of 2018, about 410,000 doses of EV-A71 vaccines (110,558 doses in 2016, 206,350 doses in 2017, and 96,900 doses in 2018) have been purchased and vaccinated by the local Centers for Disease Control (CDC) (prime and boost two-dose immunization formula). However, there was an unexpected outbreak of enterovirus infection among children in Kunming in 2018. In order to explore the situation, this study aimed to describe the clinical and epidemiology characteristics of this outbreak. Meanwhile, the incidence and epidemic characteristics of enterovirus infection were also analysed before and after EV-A71 vaccination.

## Methods

### Study design and period

This study aims to analyse the epidemic characteristics of enterovirus infection in last 11 years (2008–2018), especially 2 years before and 3 years after EV-A71 vaccines starting to use in Kunming city. Kunming Children’s Hospital, which is the unique children’s hospital in Kunming city, and it is an academic, tertiary care paediatric hospital with >1200 inpatient beds, and its total number of patients received in 2018 was 2,122,076. The information on enterovirus infection cases in Kunming Children’s Hospital was collected followed by statistical analysis from 2008 to 2018. Since an unpredicted outbreak of enterovirus infection occurred among children in Kunming in 2018, EVs positive stool samples (confirmed by real-time RT-qPCR) during the incidence of two peak periods (14 May to 29 July 2018 and 2 December 2018 to 26 January 2019) were collected from Kunming Children’s Hospital. EVs positive patients’ samples (including four clinical symptoms: Severe HFMD, HFMD, Herpangina, and fever) were harvested followed by the investigation of EVs infection spectrum, analysis of epidemiological characteristics and the relationship between clinical symptoms and EVs serotypes of infection. All patients were divided into five age groups: 6 months, 6–12 months, 1–2 years, 2–5 years and above 5 years old.

### Hospital data and stool sample collection

The information of EVs positive patients was gathered and analysed in Kunming Children’s Hospital from 2008 to 2018, EV-A71, CV-A16, and pan-EV were detected between 2008 and 2017, and the examination of CV-A10, CV-A6, and other serotypes was initiated after 2018 in this Hospital. Meanwhile, in the two peak epidemic seasons (14 May to 29 July 2018 and 2 December 2018 to 26 January 2019), 12,728 of 24,127 EVs positive (confirmed by real-time RT-qPCR in the clinical laboratory of the hospital) stool samples, including 225 Severe HFMD, 4026 HFMD, 2643 Herpangina and 5834 only fever patients’ samples were collected. Each inpatient was recorded by name, gender, number of admission, age, time of onset, sampling time, symptoms (mainly including fever: >39, vomiting, easily frightened, limb tremble, encephalitis, brainstem encephalitis, respiratory frequency, leukocyte count in peripheral blood, and circulation function: blood pressure and heart rate).

### Definitions

All 12,728 EVs positive clinical samples contained the following 4 clinical manifestations: Severe HFMD, HFMD, Herpangina, and fever. In particular, nervous system manifestations, persistent hyperthermia, abnormal breathing, and circulatory dysfunction were classified as Severe HFMD. In critically ill patients, tachycardia, shortness of breath, die blausucht, continuous decrease of blood pressure, cerebral function failure, frequent convulsions, serious disturbance of consciousness, central respiratory and circulatory failure, shock, etc.

### Laboratory testing and analysis

About 3541 of 12,728 samples were gathered in 2018, and randomly selected by Stratified Random Sampling. Accordingly, 225 Severe HFMD, 1077 HFMD, 832 Herpangina, and 1407 infectious fever patients’ sample were sent to the laboratory of the Institute of Medical Biology for sequencing and genetyping. In detail, viral RNA was extracted from stool samples, followed by reverse transcription, and then performed semi-nested PCR by designed primer [13]. Based on the VP1 region of enterovirus, universal primers were designed to obtain the genomic target fragment. The enterovirus serotype was determined by Sanger sequence and blast analysis in NCBI database [13]. Then, the EVs infection spectrum, epidemiological characteristics and the relationship between clinical symptoms and serotypes of patients were analysed.

### Statistical analysis

The chi-square test was utilized to analyse the ratio of virus subtype composition, the relationship between the number and age group of EV-A71, CV-A16, and other serotypes from 2014 to 2018, and the age distribution of mild and severe patients with hospitalized HFMD in 2018. The significant level of *p*-value was .001(two-sided) and SPSS13.0 was utilized for all data analysis.

### Ethics

This research was approved by the ethics committee of Kunming Children’s Hospital. Written consent was obtained from each participant patient’s parents or guardians in the study.

## Results

### Epidemiologyical charcteristics of HFMD cases from 2008 to 2018, before and after EV-A71 vaccine starting to use

Depending on the record of Kunming Children’s Hospital, from 2008 to 2018, the number of HFMD patients was 5893, 3626, 12,633, 8163, 12,528, 10,962, 18,595, 16,809, 14,435, 9658, and 17,008, each year. The number of severe HFMD patients was 22, 155, 782, 1003, 361, 379, 400, 657, 534, 397, and 710 annually, from 2008 to 2018. Among them, the number of critical HFMD patients was 0, 3, 9, 183, 441, 633, 1077, 735, 283, 88 and 132. Besides, the death toll of HFMD patients was 0, 1, 4, 4, 3, 3, 3, 1, 2, and 0, respectively (supplementary materials Table S1).

**Figure 1.**
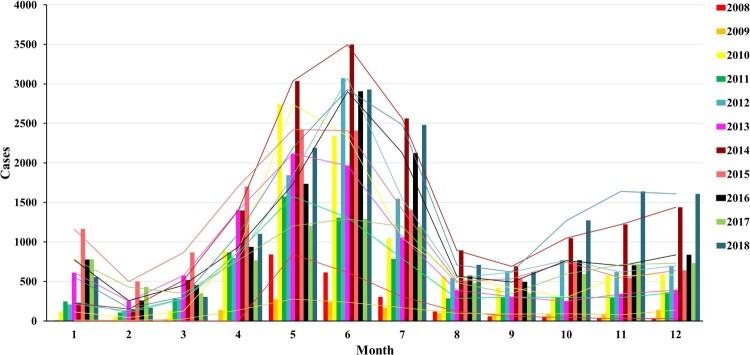
From 2008 to 2018, the monthly distribution of Hand–foot-and-mouth disease (HFMD) patients in Kunming Children’s Hospital. 18 March 2016 is the starting time point of vaccination.

The main prevalence peak of HFMD in Kunming Children’s Hospital from 2008 to 2018 was April to July. According to statistics, there is no significant change in the quantity of HFMD cases before and after EV-A71 vaccine use (Figure 1), and the epidemic peak period was almost the unanimous before and after the vaccine use. This research manifested that the majority of HFMD patients were male (supplementary materials Table S1).

### Epidemiologyical and aetiological charcteristics of enterovirus infection patients from 2014 to 2018, before and after EV-A71 vaccine starting to use

**Figure 2.**
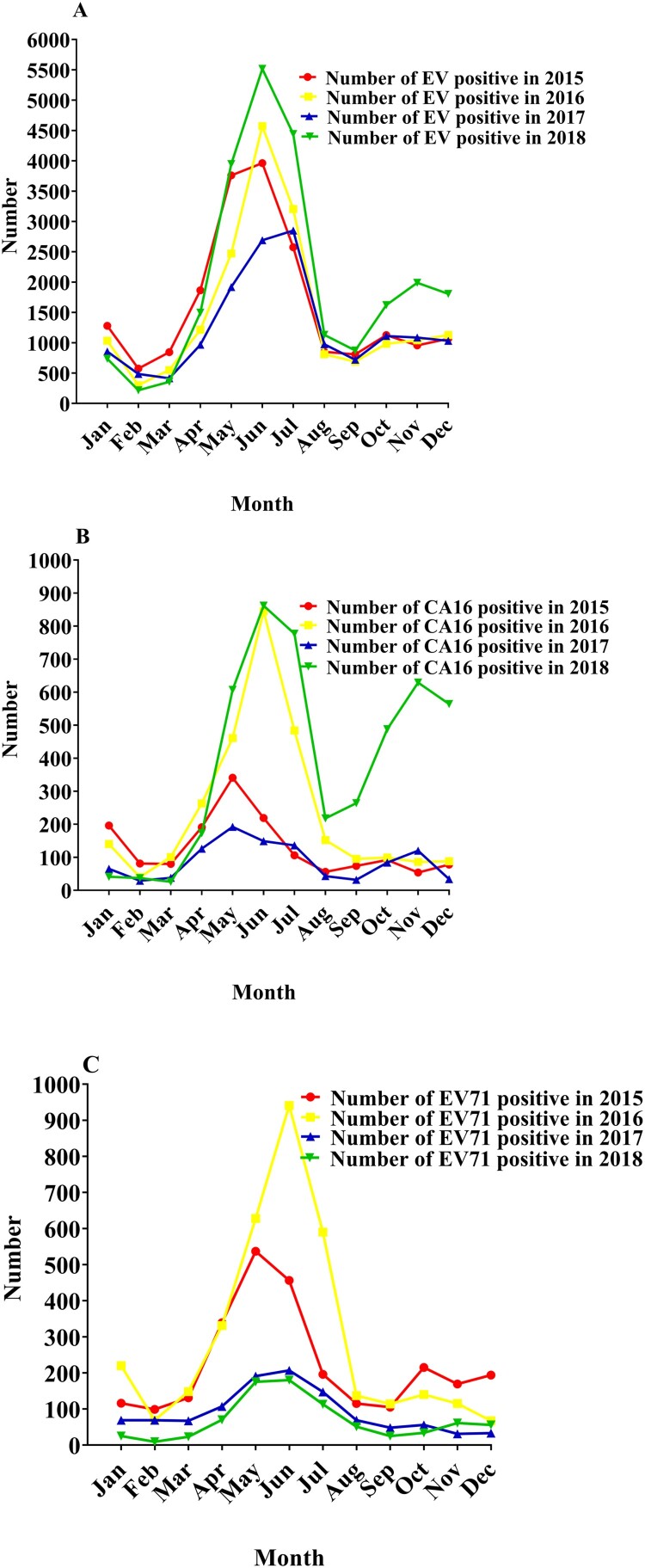
Specific statistics of Enteroviruses (EVs) positive, coxsackievirus A16 (CV-A16) and Enterovirus A71 (EV-A71) every month from 2015 to 2018 in kunming children’s hospital. (A) The specific number of EV-A71 patients per month from 2015 to 2018. (B) The specific number of CV-A16 patients per month from 2015 to 2018. (C) The specific number of EVs positive patients per month from 2015 to 2018.

The number of patients infected by enterovirus was 19,946 in 2014, 19,662 in 2015, 17,977 in 2016, 15,101 in 2017; but, it soared to 24,127 in 2018 (Figure 2(C)). The case amounts of EV-A71 infected patients were 3893 (19.52% of EVs positive), 2672 (13.59% of EVs positive), and 3500 (19.45% of EVs positive) in 2014, 2015, and 2016, respectively. After wide-ranging vaccination of EV-A71 inactivated vaccine in March 2016, the percentage of EV-A71 in total EVs infected patients decreased from 32.49% (2014), 22.30% (2015), 29.21% (2016) to 9.13% (2017) and 6.86% (2018), which was significantly decreased after EV-A71 using (Table 1).

**Table 1.** The number of enterovirus positive patients infected by Enterovirus A71 (EV-A71), coxsackievirus A16 (CV-A16), and other serotypes in Kunming Children’s Hospital from 2014 to 2018.

Years	Virus type			*P*-value^b^
	EV-A71	CV-A16	Othersa	
2014	3893 (32.49%)	4154 (29.03%)	11,899 (16.87%)	<.001^c^
2015	2672 (22.30%)	1568 (10.96%)	15,422 (21.86%)	
2016	3500 (29.21%)	2854 (19.94%)	11,643 (16.51%)	
2017	1094 (9.13%)	1048 (7.32%)	12,959 (18.37%)	
2018	822 (6.86%)	4686 (32.75%)	18,619 (26.39%)	
Total	11,981	14,310	70,542	

^a^ Enterovirus positive but non-EV-A71 and non-CV-A16.

^b^ Analysis of the difference between the constituent ratio of enterovirus types each year in 2014–2018 by chi square test.

^c^ Significant statistical differences, *P* < .001.

**Figure 3.**
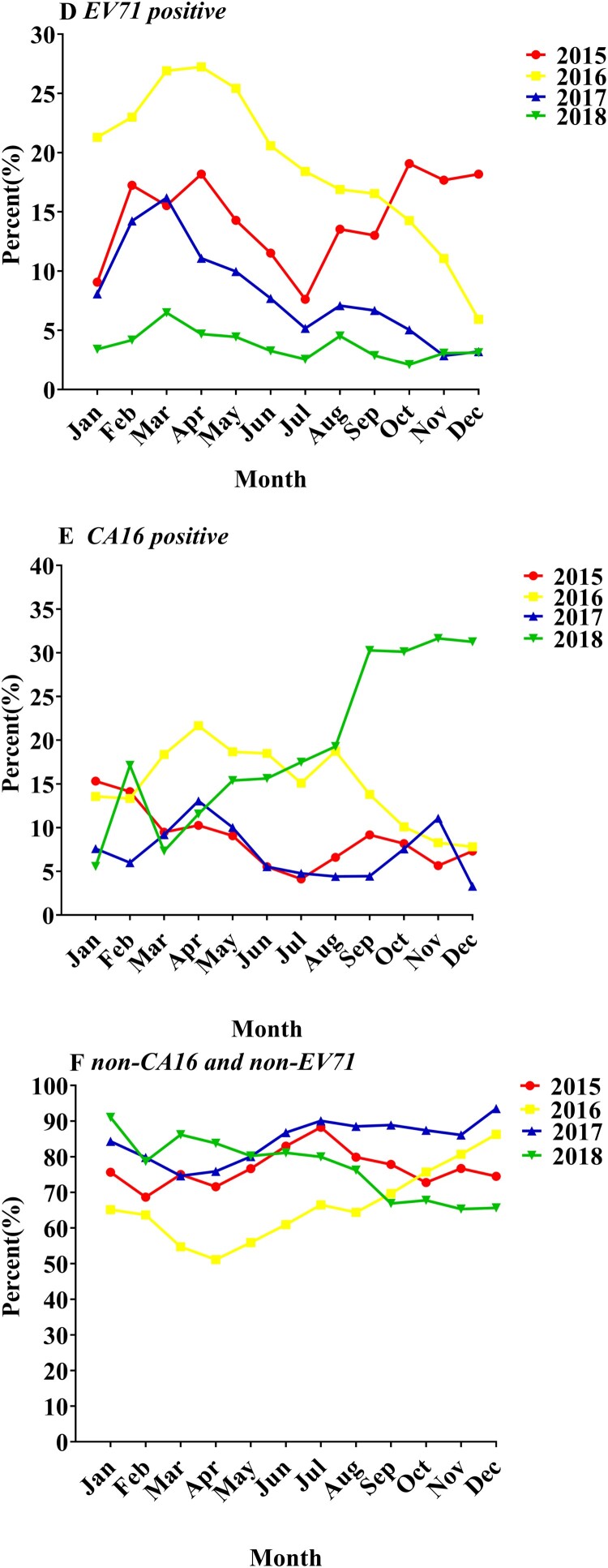
Enterovirus A71 (EV-A71), coxsackievirus A16 (CV-A16), and non-CV-A16 and non-EV-A71 as a percentage of Enteroviruses (EVs) positive per month from Kunming Children’s Hospital 2015–2018. (D) The Percentage of EV-A71 versus EVs positive per month from 2015 to 2018. (E) The Percentage of CV-A16 versus EVs positive from 2015 to 2018. (F) The percentage of non-CV-A16 and non-EV-A71 versus EVs positive from 2015 to 2018.

In addition, through the analysis of the incidence of EV-A71 infection per month in enteroviruses in 2015–2018, a significant downward trend was also observed (Figures 2(A) and 3(D)). However, compared with the case amount of 2014–2017, patients of enterovirus infection among children increased significantly in 2018, and the two peak epidemic periods of enterovirus infection were concentrated in early summer and late autumn (Figure 2(C)).

From 2014 to 2018, the amount of patients induced by CV-A16 was 4154 (20.83% of EVs positive), 1568 (8.00% of EVs positive), 2854 (15.88% of EVs positive), 1048 (6.94% of EVs positive) and 4686 (19.42% of EVs positive), respectively (Table 1). The percentage of CV-A16 patients was found to be significantly higher in 2014, 2016, and 2018, and CV-A16 infections showed a high incidence every other year (Figure 3(E), Supplementary materials Figure S1).

By analysing the characteristics of gender in enterovirus infection population from 2014 to 2018, it was denoted that there was no statistical difference (*P *> .05) between the case number of male and female patients infected by in EV-A71, CV-A16, and other subtype enterovirus each year (Table 2). The case amounts of male EV-A71 patients were 1.39, 1.39, 1.44, 1.55, and 1.43 times higher than female patients from 2014 to 2018, with an average of 1.44 times. Meanwhile, the case numbers of male CV-A16 patients were 1.35, 1.53, 1.39, 1.31, and 1.40 times higher than female patients from 2014 to 2018, with the average of 1.40 times. It manifested the same situation in other serotypes EV patients, male patients were 1.37 times higher than female patients. Meanwhile, from 2014 to 2018, patients infected with enterovirus primarily concentrated in the age range of 2–5 years and 1–2 years with the average percentage of 51.41% and 28.70% (Table 3). The age distribution of EV-A71 and CV-A16 infection patients illustrated the uniform situation (Supplementary materials Figures S2 and S3).

**Table 2.** Number of male and female patients with enteroviruses Enterovirus A71 (EV-A71), coxsackievirus A16 (CV-A16), and other serotypes in Kunming Children’s Hospital from 2014 to 2018.

	Serotype	Male	Female	Male to female ratio	*P*-value^a^
2014	EV-A71 CV-A16 Others	2267 2387 6854	1626 1767 5045	1.39 1.35 1.36	NS^b^
2015	EV-A71 CV-A16 Others	1554 949 8950	1118 619 6472	1.39 1.53 1.38	NS^b^
2016	EV-A71 CV-A16 Others	2063 1661 6733	1437 1193 4910	1.44 1.39 1.37	NS^b^
2017	EV-A71 CV-A16 Others	665 595 7511	429 453 5448	1.55 1.31 1.38	NS^b^
2018	EV-A71 CV-A16 Others	484 2733 10,733	338 1953 7886	1.43 1.40 1.36	NS^b^

^a^ Analysis of the difference between male and female in EV-A71, CV-A16 and other serotypes infected patients in 2014–2018 by chi-square test.

^b^ No statistically significant differences, *P* > .05.

**Table 3.** The relationship between age and the number of Enterovirus A71 (EV-A71), coxsackievirus A16 (CV-A16), and other serotypes in patients with enterovirus in Kunming children's Hospital from 2014 to 2018.

Age group (Years/Month)	Years					*P*-valuea
	2014	2015	2016	2017	2018	
≤0/6	365 (1.83%)	353 (1.80%)	262 (1.46%)	274 (1.81%)	412 (1.71%)	<.001^b^
0/6–0/12	1573 (7.89%)	1861 (9.46%)	955 (5.31%)	1544 (10.22%)	1970 (8.17%)	
1/0–2/0	5812 (29.14%)	5872 (29.86%)	4696 (26.09%)	4131 (27.36%)	7484 (31.02%)	
2/0–5/0	10,303 (51.65%)	9976 (50.74%)	10,241 (56.90%)	7665 (50.76%)	11,340 (47.00%)	
5/0–14/0	1847 (9.26%)	1595 (8.11%)	1835 (10.20%)	1481 (9.81%)	2901 (12.02%)	
≥14/0	46 (0.23%)	5 (0.03%)	8 (0.04%)	6 (0.04%)	20 (0.08%)	
Total	19,946	19,662	17,997	15,101	24,127	

^a^ Analysis of the difference between the number and age group of EV-A71, CV-A16, and other serotypes in 2014–2018 by chi-square test.

^b^ Significant statistical differences, *P* < .001.

### Disease symptoms and serotype distribution of enterovirus infection in 2018, 2 years after EV-A71 vaccine starting to use

The number of EVs positive patients in 2018 (24,127 cases) was significantly greater than that in the previous four years in Kunming City. To further investigate serotype distribution of enterovirus in diverse disease symptoms, 225 stool samples from severe HFMD patients (225 cases), 1077 stool samples from HFMD patients (4026 cases), 832 stool samples from Herpangina patients (2643 cases) and 1407 stool samples from patients with infectious fever (5834 cases) were serotyped in a laboratory using semi-nested PCR. About 146 of 225 (64.89%) severe HFMD samples, 725 of 1077 (67.32%) HFMD samples, 557 of 832 (66.95%) Herpangina samples, and 985 of 1407 (70.01%) infectious fever stool samples were successfully typed (Supplementary materials Table S2). Twenty-two serotype enteroviruses were detected, including 10 coxsackievirus type A, 4 coxsackievirus type B, 7 echoviruses, and enterovirus 71 (Supplementary materials Figure S4).

Among all clinical symptoms, CV-A6 had the highest infection rate, accounting for 62.33%, 52.41%, 43.98%, and 42.13% in severe HFMD, HFMD, HA, and Fever, respectively, followed by CV-A10 or CV-A16 (Supplementary materials Table S2). It is worth mentioning that CV-A6 accounting for 62.33% in severe HFMD. CV-A6, CV-A10, and CV-A16 become the major serotypes of enterovirus infection in Kunming, 2018. Previous studies have also manifested that CV-A10 belongs to the enterovirus A (EV-A) species and has been identified as the major pathogen during recent outbreaks in Asia and Europe, along with other members such as EV-A71, CV-A16, and CV-A6 [15–19]. In addition to CV-A6 and CV-A10, we discovered a significant increase of CV-A4 in all clinical disease symptoms, ranking fourth in all clinical symptoms (12.59% in Fever, 10.41% in HA, 1.79% in HFMD, and 5.48% in severe HFMD). There also indicate that the infection of CV-A4 cannot be underestimated.

### Serotype distribution of enterovirus in hospitalized HFMD patients collected in 2018, 2 years after enterovirus 71 inactivated starting to use

According to the information of hospitalized HFMD patients collected in 2018, the quantity of boys (145/252, 57.54%) was about 1.36 times that of girls (107/252, 42.46%). It is elucidated that there is no difference (*P *> .05) in gender composition between mild HFMD and severe HFMD patients, while there were changes in age composition (*P* < .001). The majority of severe HFMD patients were between 1 and 2 years old (60.44%), while the majority of mild HFMD patients were between 2 and 5 years old (48.15%). Besides, 10 patients were co-infected by 2 diverse serotypes of enterovirus, including 2 patients co-infected by CV-A6 and CV-A10, 4 patients by CV-A6 and CV-A16, 2 patients by CV-A6 and CV-A4, 1 patient by CV-A6 and CV-B5, 1 patient by CV-A16 and EV-A71.(Table 4).

**Table 4.** Epidemiological of severe Hand-foot-and-mouth disease (HFMD) patients that were admitted to Kunming Children’s Hospital (*N* = 252).

	Mild HFMD patients/total	Severe HFMD patients/total	*P*-value^a^
Sex			NSb
Female	10/27 (37.04%)	97/225 (43.11%)	
Male	17/27 (62.96%)	128/225 (56.89%)	
Age group (years/month)			<.001^c^
≤0/6	2/27 (7.41%)	2/225 (0.89%)	
0/6–0/12	6/27 (22.22%)	18/225 (8.00%)	
1/0-2/0	5/27 (18.52%)	136/225 (60.44%)	
2/0–5/0	13/27 (48.15%)	56/225 (24.89%)	
>5/0	1/27 (3.70%)	13/225 (5.78%)	
Co-infection			–^d^
CV-A6 / CV-A10	–	2/252 (0.89%)	
CV-A6 / CV-A16	1/27 (3.70%)	4/252 (1.78%)	
CV-A6 / CV-A4	–	2/252 (0.89%)	
CV-A6 / CV-B5	–	1/252 (0.44%)	
CV-A16 /EV-A71	–	1/252 (0.44%)	

^a^ Comparison of mild and severe cases of hospitalized HFMD with sex and age group by chi-square test.

^b^ No statistically significant differences, *P* > .05.

^c^ Significant statistical differences, *P* < .001.

^d^ Since there were 0 cases of co-infection in mild cases, no statistics were performed.

Among the severe HFMD patients gathered in 2018, the dominating symptoms of the patients were encephalitis (90/225, 40.00%), persistent hyperthermia (body temperature >39°C (128/225, 56.89%)), vomiting (47/225, 20.89%), ease of being startled (117/225, 52.00%), limb tremors (43/225, 19.11%) (supplementary materials Table S3), among which, 8 out of 225 severe patients were critical HFMD, and half of the critically ill patients had brainstem encephalitis.

## Discussion

HFMD is a global disease. It was first recognized and generally named based on its clinical characteristics in New Zealand [20] in 1957. In the past two decades, HFMD has become popular in the Asia-Pacific region, including Malaysia, Taiwan, Japan, Singapore, Vietnam, Hong Kong, Korea and Cambodia [21–23]. It was discovered in 1981 in China, HFMD occurs all over China throughout the year, with an incidence of 37.01/100,000–205.06/100,000, and a reported case fatality rate of 6.46/100,000–51.00/100,000 in recent years. It is estimated that the annual cost (direct medical cost and indirect cost) of severe and mild HFMD cases caused by EV-A71 infection were about 25 million and 140–280 million U.S. dollar, respectively. The reported incidence of HFMD in China has also been on an upward trend in recent years, while the incidence of severe disease and death has decreased, and the reported incidence of severe disease and death has been characterized by a high incidence every other year. In 2017, a total of 7,030,879 cases of statutory infectious diseases were reported nationwide, with 19,796 deaths. Of these statutory infectious diseases, 27.4% (1,929,550/7,030,879) were HFMD cases with mortality rate of 0.48% (95/19,796). However, the number of HFMD patients reached 2,375,938 in China, 2018. From 2009 to 2018, the information of China CDC showed that HFMD presented a dynamic change with a high incidence every two years [24]. Our data on EVs positive patients from 2015 to 2018 also showed that the incidence of children’s HFMD was high every other year in Kunming city.

EV-A71 is the primary serotype in severe and fatal cases, and the other enterovirus components in severe cases increased after 2013. In 1969, EV-A71 was first isolated from stool specimens of infants with symptoms of central nervous system (CNS) infection in California, USA [25]. In 1998, EV-A71 was isolated from the samples of HFMD children in Shenzhen, China for the first time. Since 2007, EV-A71 infection-related diseases have been widespread in China [26]. On 18 March 2016, the inactivated enterovirus 71 vaccine (human diploid cells), developed by the Institute of Medical Biology of the Chinese Academy of Medical Sciences, was officially put on the market. Till the end of 2018, about 410,000 doses of EV-A71 vaccines (110,558 doses in 2016, 206,350 doses in 2017, and 96,900 doses in 2018) have been purchased and vaccinated by local centres for Disease Control (CDC) (prime and boost two-dose immunization formula). As a medium-sized city, Kunming has a population of 6.783 million in 2018; including 1.08 million children aged 0–14, accounting for nearly one sixth of the total population. According to the dose of vaccines purchased and immunized, nearly 19.16% of children have been vaccinated in Kunming city. However, there was an unpredicted outbreak of enterovirus infection among children in Kunming in 2018. Clinical data collected by Kunming Children’s Hospital (the unique children's hospital in this city) elucidated that the number of EV-A71 positive patients in 2017 (1094 cases) and 2018 (822 cases) dropped sharply compared with 2015 (2672) and 2016 (3500). It was reported that EV-A71 circulates in 3–4 year cycles in Japan and Malaysia and CV-A16 circulates in a cyclical pattern of every 2–3 years in Singapore, England, and Wales [27]. However, the prevalence interval in most areas of China is mostly one year, and some provinces have been observed the characteristics of the periodic epidemic in 2–3 years. Our clinical follow-up data of nearly four years manifested that the application of EV-A71 vaccine may play a significant effect in children of Kunming. In addition, because immunization coverage against EV-A71 is not high enough for herd immunity in Kunming city, the changes in the main types of enterovirus infection and the increase in parental awareness in recent years are also likely to be a potential factor in the incidence rate of EV-A71 infection. Meanwhile, we were more concerned about whether there were cases of EV-A71 patients who had been vaccinated with the EV-A71 vaccine before. In fact, we observed two cases of HFMD caused by EV-A71 after vaccination of EV-A71 inactivated vaccine. The possible reason was amino acid mutations occurred in EV-A71 isolates of these two patients or individual difference. Considering the cyclical transmission of EV-A71, continuous monitoring is requisite to facilitate the effectiveness of vaccine prevention and protection.

CV-A16 patients presented a high incidence every other year; especially the incidence in 2018, and was almost doubled compared with previous years. This phenomenon may be attributed to the increasing popularity of HFMD. Besides, the EV-A71 vaccine has no protective effect against CV-A16 and other enterovirus infection [28,29]. Although the significant effect of the EV-A71 vaccine has been confirmed in the follow-up investigation of Kunming city in recent year, how large-scale application of the EV-A71 vaccine will promote serotype change of infection is still concerned. Our laboratory results revealed that CV-A6, CV-A10 and CV-A16 are the predominant types of viruses in the stool sample of all EVs positive patients in Kunming (Southwest China), 2018. Meanwhile, according to the test results of Shandong (East China) and Shenzhen (Southeast China) samples collected by our laboratory in 2018, of the 8 samples originated from Shandong Province, CV-A6 and CV-A10 were 6 and 2, respectively; of the 10 samples derived from Shenzhen city, 6 were CV-A 6 and 4 were CV-A10. In the recent years, sporadic cases and outbreaks of HFMD have been widely associated with CV-A6 and CV-A10, particularly in South East Asia, India and Europe, with an increased risk of neurological complications and mortality [30].

Depending on the record of Kunming Children’s Hospital from 2008 to 2018, although the amount of HFMD patients will fluctuate every year, the overall mortality rate shows a downward trend, which may be closely related to the safety awareness of the parents and the improvement of the medical level of the hospital. In addition, although HFMD broke out in 2018, to some extent, it is perhaps associated with the improvement of detection level and limit, the enhancement of transportation and the opening of the national two-child policy to promote the increase of neonatal birth rate.

The EV-A71 vaccine only has a good protective effect against EV-A71 but has little effect on the overall enterovirus epidemic. In addition, the large-scale application of the vaccine may promote serotype transformation to some extent. Although some of the deficiencies exist in our research, the effect of EV-A71 vaccine and the changes of main serotypes of enterovirus causing HFMD after its use was studied. At the same time, the direction of vaccine development in the future and global prevention are suggested. Our results suggest that EV-A71 inactivated vaccine has a very good protective effect against EV-A71. After using of EV-A71 vaccine, EVs infection spectrum has changed. According to our limited research results, EV-A71 vaccine has no or poor cross-protection effect on CV-A6, CV-A10, and CV-A16. The research and development of 4-valent combined vaccines including CV-A6, CV-A10, CV-A16, and EV-A71 are urgently needed.

## Supplementary Material

Clean_copy_Supplementary_materials.doc
