# Supplementary material for: The epidemiological characteristics of enterovirus infection before and after the use of enterovirus 71 inactivated vaccine in Kunming, China
Source: Emerg Microbes Infect. 2021 Mar 30;10(1):619–28. doi: 10.1080/22221751.2021.1899772 (PMC8018479; doi:10.1080/22221751.2021.1899772)
Supplement: Clean_copy_Supplementary_materials.doc [file TEMI_A_1899772_SM0402.doc]

**Table S1 The number of Hand-foot-and-mouth disease (HFMD) patients in Kunming children's Hospital from 2008 to 2018.**

| **Year** | **Outpatients** | **Hospitalized patients** | **Severe patients** | **Critical patients** | **Deaths** |
| --- | --- | --- | --- | --- | --- |
| 2008 | 5733 | 160 | 22 | 0 | 0 |
| 2009 | 3326 | 300 | 155 | 3 | 1 |
| 2010 | 11329 | 1304 | 782 | 9 | 4 |
| 2011 | 6714 | 1449 | 1003 | 183 | 4 |
| 2012 | 11131 | 1397 | 361 | 441 | 3 |
| 2013 | 9550 | 1412 | 379 | 633 | 3 |
| 2014 | 16723 | 1872 | 400 | 1077 | 3 |
| 2015 | 15088 | 1721 | 657 | 735 | 1 |
| 2016 | 13313 | 1122 | 534 | 283 | 2 |
| 2017 | 8885 | 773 | 397 | 88 | 0 |
| 2018 | 15584 | 1424 | 710 | 132 | 1 |

**Table S2 Laboratory Results: The virus types and percentage of Hand, foot and mouth disease (HFMD), and Herpangina (HA), Others (Infections fever) collected from Kunming Children's Hospital during May 14, 2018 to July 29, and December 02, 2018 to January 26, 2019 (All samples were EV positive). N: represents the ratio of the number of patients with successful enterovirus typing to the selected sample.**

| Others (Infectious fever)  **（N=985/1407,70.01%）**  Type No.(%) | HA  **（N=557/832,66.95%）**  Type No.(%) | HFMD  **（N=725/1077,67.32%）**  Type No.(%) | Severe HFMD  **（N=146/225,64.89%）**  Type No.(%) |
| --- | --- | --- | --- |
| CV-A6 **415(42.13)**  CV-A16 **191(19.40)**  CV-A10 **178(18.07)**  CV-A4 **124(12.59)**  EV-A71 **13(1.32)**  CV-A9 **9(0.91)**  CV-B5 **8(0.81)**  ECO11 **6(0.61)**  CV-A2 **5(0.51)**  CV-A12 **4(0.41)**  CV-A5 **4(0.41)**  ECO5 **4(0.41)**  CV-A8 **3(3.06)**  ECO18 **3(0.31)**  CV-B4 **3(0.31)**  CV-B3 **2(0.20)**  ECO9 **2(0.20)**  ECO6 **2(0.20)**  ECO16 **1(0.10)**  ECO25 **1(0.10)**  CV-A6 and CV-A10 **1(0.10)**  CV-A10 and CV-B5 **1(0.10)**  CV-A16 and CV-A9 **1(0.10)**  CV-A16 and CV-B5 **1(0.10)**  CV-A4 and CV-B5 **1(0.10)**  CV-A6 and CV-B1 **1(0.10)**  CV-A10 and ECO5 **1(0.10)** | CV-A6 **245(43.98)**  CV-A10 **133(23.9)**  CV-A16 **101(18.13)**  CV-A4 **58(10.41)**  CV-A5 **8(1.44)**  EV-A71 **2(0.36)**  CV-A12 **2(0.36)**  CV-B5 **2(0.36)**  CV-A6 and CV-B5 **2(0.36)**  CV-A2 **1(0.18)**  CV-A14 **1(0.18)**  CV-A10 and CV-B5 **1(0.18)**  CV-A6 and CV-A4 **1(0.18)** | CV-A6 **380(52.41)**  CV-A16 **257(35.45)**  CV-A10 **50(6.90)**  CV-A4 **13(1.79)**  CV-A16 and EV-A71 **7(0.97)**  EV-A71 **5(0.69)**  CV-A5 **2(0.28)**  CV-A6 and CV-A16 **2(0.28)**  CV-A6 and EV-A71 **2(0.28)**  CV-A12 **1(0.14)**  CV-A8 **1(0.14)**  CV-B5 **1(0.14)**  CV-B4 **1(0.14)**  ECO11 **1(0.14)**  CV-A6 and CV-A10 **1(0.14)**  CV-A10 and CV-A16 **1(0.14)** | CV-A6 **91(62.33)**  CV-A10 **17(11.64)**  CV-A16 **16(10.96)**  CV-A4 **8(5.48)**  EV-A71 **3(2.05)**  ECO5 **2****(1.37)**  CV-A6 and CV-A4 **2(1.37)**  CV-A6 and CV-B5 **2(1.37)**  CV-A6 and CV-A10 **2(1.37)**  CV-A12 **1(0.68)**  CV-A6 and CV-A16 **1(0.68)**  CV-A16 and EV-A71 **1(0.68)** |

**Table S3 Analysis of the clinical characteristics of severe hospitalized Hand-foot-and-mouth disease (HFMD) patients collected in Kunming children's Hospital in 2018 (N=225).**

| **Severity of symptoms** | **NO. (%)** |
| --- | --- |
| Severe case | 217/225(96.44%) |
| critical case | 8/225(3.56%) |
| **Symptoms** |  |
| Persistent hyperthermia (>39℃) | 128/225(56.89%) |
| Vomit | 47/225(20.89%) |
| Ease of being startled | 117/225(52.00%) |
| Limb tremors | 43/225(19.11%) |
| Vomit and Hyperarousal | 29/225(12.89%) |
| Vomit and Limb tremble | 12/225(5.33%) |
| Hyperarousal and Limb tremble | 24/225(10.67%) |
| Vomit,Hyperarousal and Limb tremble | 7/225(3.11%) |
| Encephalitis | 90/225(40.00%) |
| Brainstem encephalitis | 6/225(2.67%) |
| Breathing accelerated (>40/min) | 1/225(0.44%) |
| Heart rate increased (>160/min) | 6/225(2.67%) |
| Peripheral white blood cell count increased (≥15×109/L) | 24/225(10.67%) |

**
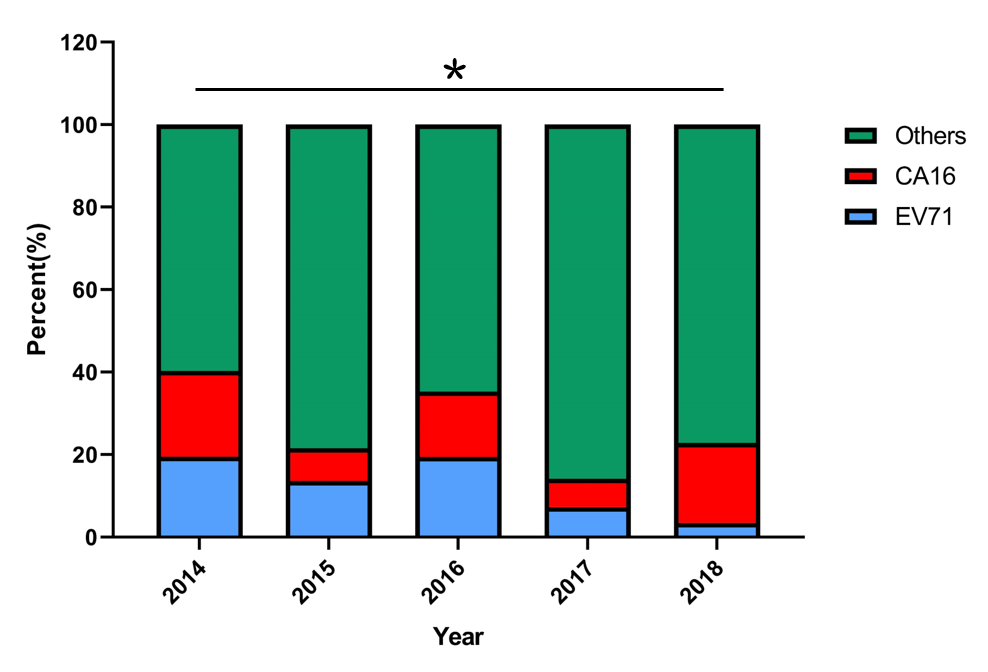
**

**Figure S1 Percentage of non-CV-A16 and non-EV-A71, Enterovirus A71 (EV-A71), coxsackievirus A16 (CV-A16) enterovirus patients in Kunming Children's Hospital from 2014 to 2018**. ^*^ **P<0.001** **by chi square test.**





**Figure S2 Incidence and age distribution of Enterovirus A71 (EV-A71) and coxsackievirus A16 (CV-A16) patients in Kunming Children's Hospital from 2015 to 2018.**

1. Distribution of EV-A71 patients in disparate age groups from 2015 to 2018 (J) Distribution of CV-A16 patients in different age groups from 2015 to 2018.

**

**

**Figure S3 Male-to-female ratio of coxsackievirus A16 and Enterovirus A71 (EV-A71) patients in Kunming Children's Hospital from 2014 to 2018.** (U) The sex ratio of EV-A71 patients in recent four years. (V) The sex ratio of CV-A16 patients in recent four years.

**
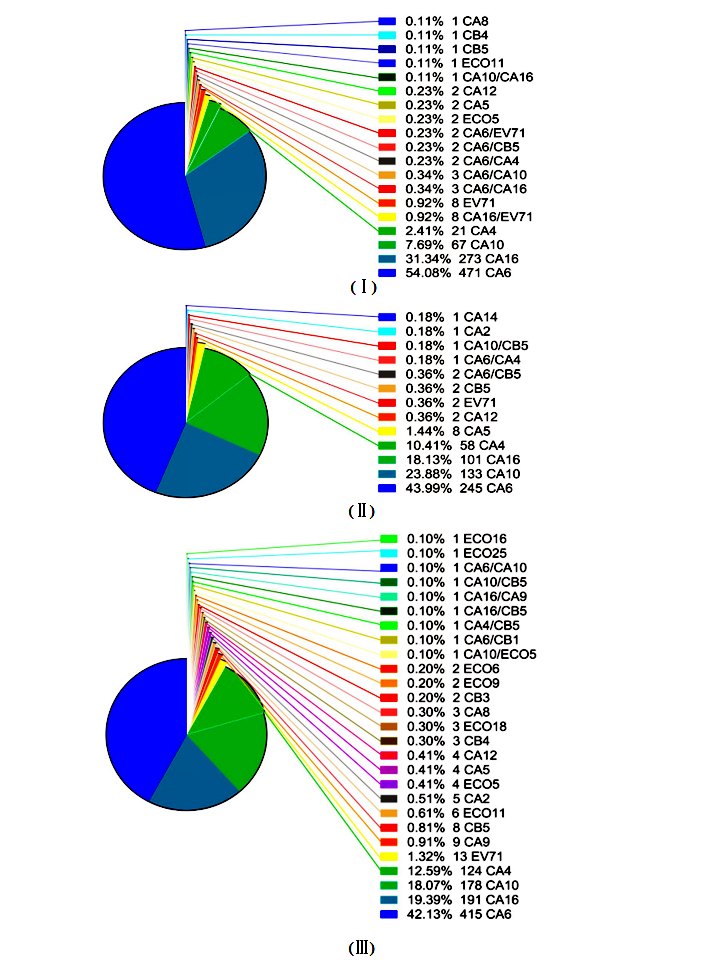
**

**Figure S4**  **Comparison of laboratory typing results of stool samples at the two peak periods.** (Ⅰ) Proportion of laboratory typing results for Hand-foot-and-mouth disease (HFMD) patients (871) during May 14, 2018 to July 29, and December 02, 2018 to January 26, 2019. (Ⅱ) Proportion of laboratory typing results for HA patients (557) during May 14, 2018 to July 29, and December 02, 2018 to January 26, 2019. (Ⅲ) Proportion of laboratory typing results of others patients [Infectious fever (985)] during May 14, 2018 to July 29, and December 02, 2018 to January 26, 2019.

**
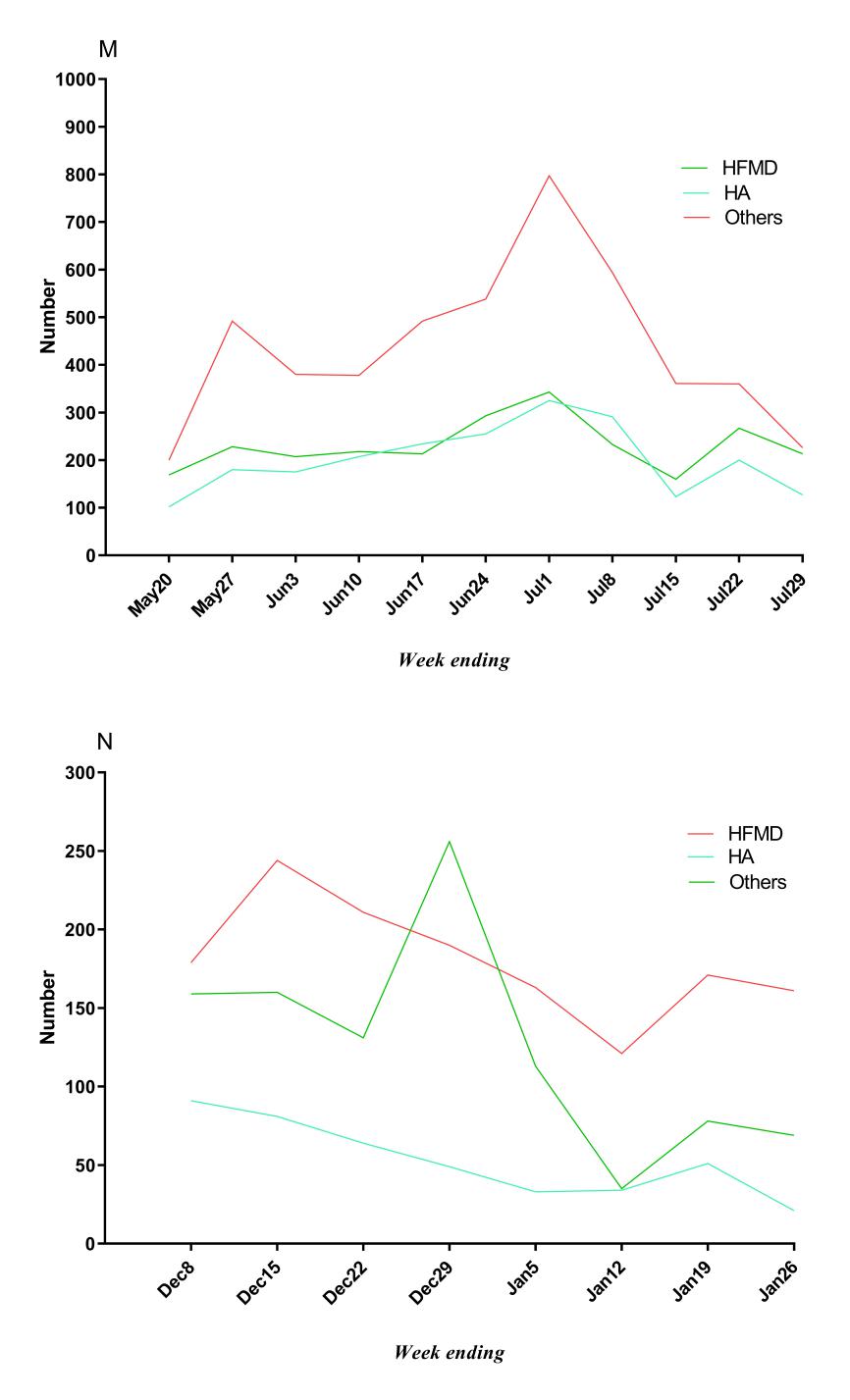
**

**Figure S5 Specific situation of outpatient enterovirus patients gathered from Kunming Children's Hospital during two peak periods.**

1. Visits to the sample collection in children with enterovirus during May 14, 2018 to July 29, and (N) Visits to the sample collection in children with enterovirus during December 02, 2018 to January 26, 2019.
